# Multimorbidity and leisure-time physical activity over the life course: a population-based birth cohort study

**DOI:** 10.1186/s12889-021-10719-7

**Published:** 2021-04-09

**Authors:** Natan Feter, Jayne S. Leite, Daniel Umpierre, Eduardo L. Caputo, Airton J. Rombaldi

**Affiliations:** 1 Postgraduate Program in Physical Education, Universidade Federal de Pelotas, Rua Luís de Camões, 625, Pelotas, 96055630 Brazil; 2 GEEAF - Physical Activity Epidemiology Research Group, Universidade Federal de Pelotas, Rua Luís de Camões, 625, Pelotas, 96055630 Brazil; 3 Centre for Research on Exercise, Physical Activity and Health (CRExPAH), School of Human Movement and Nutrition Sciences, The University of Queensland, St Lucia, QLD 4067 Australia; 4 Postgraduate Program in Health Sciences, Universidade Federal do Rio Grande do Sul, Porto Alegre, RS Brazil; 5 Health Technology Assessment Institute, Clinical Research Center, Hospital de Clínicas de Porto Alegre, Porto Alegre, RS Brazil; 6 Department of Public Health, Universidade Federal do Rio Grande do Sul, Porto Alegre, RS Brazil

**Keywords:** Physical activity, Multimorbidity, Cohort, Life course

## Abstract

**Background:**

We aimed to test which life course model best described the association between leisure-time physical activity (LTPA) and multimorbidity at age 55. We analyzed data from birth to age 55 using the database from the 1958 National Child Development Survey.

**Methods:**

Multimorbidity was considered as the presence of more than one chronic condition. LTPA was measured through questionnaires from 1965 (age 7) to 2013 (age 55), which were applied in eight different occasions. We compared the fit of a series of nested adjusted logistic regression models (representing either the critical, accumulation or sensitive period models) with a fully saturated model. Data were reported as odds ratio (OR) and 95% confidence interval (CI).

**Results:**

From an eligible sample of 15,613 cohort members, 9137 were interviewed in the latest sweep (58.5%). Men were more physically active than women at ages 11, 16, and 23 (*p* < 0.001). LTPA every day in the week was more frequent in women than men in ages 33, 42, and 50 (*p* < 0.001). The prevalence of multimorbidity at age 55 was 33.0% (*n* = 2778). The sensitive analysis revealed that LTPA during adolescence (OR: 0.83; 95% CI: 0.70, 0.98) and mid adult life (age 50 and 55; OR: 0.82; 95%CI: 0.69, 0.98) have a stronger effect on the risk for multimorbidity at age 55 considering all other life stages in the model. Also, adolescence showed a critical independent effect on the risk for multimorbidity (OR: 0.82; 95%CI: 0.70, 0.97). No difference was found between those models.

**Conclusions:**

These data support the notion of a protective physical activity “legacy” at early ages of childhood against multimorbidity at older ages. We highlight the need for LTPA promotion through intervention tailored especially on schooling and older ages in order to reduce the burden of multimorbidity.

**Supplementary Information:**

The online version contains supplementary material available at 10.1186/s12889-021-10719-7.

## Introduction

Multimorbidity, defined as the co-occurrence of two or more chronic conditions in the same individual [1], affects adults from all age groups, and its burden increases with aging [2]. It is associated with diminished quality of life of both individuals and their families [3]. Likewise, people with multimorbidity have higher frequency of hospitalizations [4, 5], which are usually followed by decreased functional capacity, cognitive function, and increased use of prescribed medications [6]. One important clinical aspect of multimorbidity is its multifactorial etiology. The chronic conditions that are affecting the same individual simultaneously are related to an impairment on different organic systems, which may lead to higher risk of disability and mortality [7–9]. Also, the burden of multimorbidity for the patient is increased when primary care systems fail to appropriately address the needs of these patients [9, 10].

Moreover, multimorbidity is associated with increased risk of all-cause mortality in a dose-response effect [11] regardless of age, gender, and economic stratus [12]. Therefore, investigations about how lifestyle factors through life course could be associated with multimorbidity in older ages should be encouraged. Physical activity should be considered an alternative that deserves attention and investment, given its benefits on prevention and treatment of several noncommunicable chronic diseases [13].

Physical activity can be divided into four domains: leisure-time, occupational, commuting, and household activities. Leisure-time physical activity (LTPA) is related to physical activities performed during spare hours or leisure periods, based on individual or collective preferences. LTPDA has been shown an inverse relationship with all-cause, specific-cause mortality [14], and unhealthy ageing (i.e., ageing with no chronic conditions, cognitive impairment, and physical dependency) [15]. However, it is still scantly explored whether (and at which extent) LTPA in early ages or over different life stages could be associated with different rates of non-communicable disease in later life. Our hypothesis is that people who were physically active in early life stages would have a lower risk for multimorbidity than those with low physical activity practice.

Thus, the present study aimed to assess whether there is an association of LTPA at different stages of life with multimorbidity at age 55 and evaluate its cumulative effect during the early life stages on multimorbidity in middle-to-older adults.

## Methods

### Study design

We analyzed data from the 1958 National Child Development Survey (NCDS). The dataset is freely available upon request at the UK Data Service [16] (https://www.ukdataservice.ac.uk/deposit-data/). Full description of sampling design and methods can be found elsewhere [17]. Briefly, this birth cohort collected data from approximately 94% (*N* = 17,638) of all births between 3rd and 9th March 1958 in England, Wales, and Scotland. Sociodemographic and behavioral information from both parents besides data regarding to pregnancy and the child were assessed. Further, any child born in the Great Britain in that specific week were identified by school registers and added to cohort sample during second through fourth sweeps (1965, 1969, and 1974; *n* = 920), reaching a final sample of 18,558 cohort members. Flow diagram about sample composition is shown in Figure S1 (supplementary material).

Fifty-five years after baseline assessments, this cohort remains largely representative of the sample that it was drawn. In the latest sweep (2013) cumulative deaths (*n* = 1659) and emigrants (*n* = 1286) through cohort summed 2945. Thus, from an eligible sample of 15,613, 9137 participants were interviewed (response rate: 58.5%) [17]. After baseline measurements, new sweeps occurred in 1965 (cohort age: 7 years), 1969 (11 years), 1974 (16 years), 1981 (23 years), 1991 (33 years), 2000 (42 years), 2002 (44 years), 2004 (46 years), 2008 (50 years), and the latest in 2013 (55 years). The 2002 and 2004 sweeps were not included in the present analysis due to methodological distinctions compared to the others (i.e. self-reported and telephone-based interview, respectively). The next sweep was programed to occur in 2020 and 2021; however, fieldwork was paused in light of COVID-19. The full access to all questionnaires used during follow-up assessments can be found elsewhere (https://cls.ucl.ac.uk/cls-studies/1958-national-child-development-study-2/).

### Outcome

We considered multimorbidity as the co-occurrence of more than one of the following morbidities at age 55 [1]: asthma or wheezy bronchitis, cancer, backache, depression, diabetes, coronary heart disease, high blood pressure, obesity (self-reported body mass index ≥30.0), and visual and hearing impairment. Then, based on this classification, a dichotomous variable was created.

### Exposure

LTPA was assessed from 1965 to 2013. Data collected in 2002 (age 44 sweep) and 2004 (age 46 sweep) regarding to LTPA were not used in this study because they were assessed by different interview methods (self-administrated and telephone-based interview, respectively), which may decrease significantly both reliability and comparability of measurements [18]. All included LTPA data were assessed by face-to-face interview-administered paper-based questionnaire. Supplementary Table S1 (electronic supplemental material) describes how it was measured in each sweep and how we operationalized it for this study. Briefly, participants were dichotomously classified as physically active when performed LTPA regularly (age 7, 11, and 16) and at least once per week (from age 23 to the latest), as in previous work [19]. Participants who practiced LTPA in level lower than these, they were classified as physically inactive. For age 7, LTPA was assessed based on mother’s perception of how active was active during the day (inactive, normally active, over active). For age 11, the children were asked how often (never or hardly ever, sometimes, often) they played outdoor games or take part in sports outside of school hours. For age 16, a list of indoor and outdoor activities was provided to participants, so they could report the frequency in each activity (not available, never, sometimes, often). Often was scored as 2, sometimes scored as 1, never and not available were scored as 0. The scores were summed across the variables and the resulting categories collapsed to 4. We dichotomized the resulting variable as active (the two most active categories) and inactive (the two least active). For age 23 and older, participants were considered physically active if performed LTPA once a week or more and inactive if practiced LTPA less than once a week. Full description of LTPA operationalization is provided as Supplementary Table S1 and in previous publications [20].

### Confounding variables

All multivariate analyses were adjusted for the following variables: gender, marital status, education level, income, country of birth, ethnicity, smoking, alcohol intake, hours of sleep, and LTPA. We considered sex, country of birth, and ethnicity collected at birth sweep. We used LTPA as a possible confounder in analysis where the main exposure was a time-specific sweep and it was adjusted for previous and future LTPA practice. All remaining used variables were assessed in the latest sweep (2013).

### Statistical analysis

Descriptive analysis is reported as absolute and relative frequencies. The difference between groups was assessed using chi-square tests. To evaluate the PA effect during earlier life, we stratified the variable in childhood (age 7 and 11 sweeps), adolescence (age 16 sweep), young (age 23 sweep), middle (age 33 sweep), and middle-to-old age (age 50 and 55 sweeps) adults.

Logistic regression was performed as crude analysis (model 1) and using hierarchical model adjusting for gender, marital status, education level, income, country of birth, ethnicity (model 2), as well as smoking, alcohol intake, and hours of sleep (model 3). For regression analyses, PA was categorized as either “inactive” or “active” for each individual. Such categorization was conducted for each life stage considered in the analysis and was based on the daily or weekly frequency of PA. The criteria used for PA categorization for each life stage are presented in Supplementary Table S1.

Then, a structured modeling approach developed by Mishra et al. [21] was used to select the most appropriate life course model for multimorbidity at age 55. This method allows to determine how the changes in PA over the life course might attenuate the risk of multimorbidity in middle-to-late adulthood. Four hypothesized life course models were examined: saturated, critical, sensitive, and accumulation. Saturated model included all possible exposure combinations and interactions and describes all possible trajectories of LTPA throughout life course (childhood, adolescence, young, middle, and middle-to-older adults).

Accumulation model was tested in two versions. First, a strict model (continuous) was assessed by adding the number of times an individual reported being physically active across their life course to form an overall score, which was then used as the exposure. This model assumes that the effect of LTPA at each life period (childhood, adolescence, adulthood) period is the same. Second, a relaxed model (categorical) was examined in which all time periods are contributing to multimorbidity at age 55 but not necessarily in an equal way.

The critical period model assumes that only LTPA in a certain age influences multimorbidity at age 55 regardless of any other time period. Similarly, sensitive model was tested to allow the examination of the varied effect of LTPA across the life course, which can be modelled by simultaneously including all LTPA variables in the model. Finally, a null model was tested with only our outcome at the model [21].

To identify the most appropriate life course model to explain multimorbidity at age 55, likelihood ratio test was conducted comparing each life course model to the saturated model. When nested life course models (critical period, sensitive period, and accumulation) provided similar fit to the fully saturated model (*p* > 0.05), the one with the lowest Akaike’s information criterion (AIC) was selected. When more than one model presented *p*-value higher than 0.05 and there is not a large difference in *p*-values, the simpler model was selected [21].

To minimize data loss, missing data were imputed using multiple imputation chained equations as recommended by the NCDS user guide [22]. We ran imputation models with all variables from our logistic models across 20 imputed datasets. As imputed results were similar to those obtained using observed values, the latest was presented. All statistical analyzes were carried out using STATA 13.1 software (StataCorp, College Station, Texas). A *p* value of < 0.05 was accepted as statistically significant.

## Results

From the initial sample of 18,558 cohort members, 9137 (49.2%) were interviewed in the latest sweep. Excluded participants were more likely to be male (54.8%), have no academic qualification (36.8%), be divorced/separated (24.7%), unemployed (16.5%), and smoker at age 50 (35.9%), as shown in Supplementary Table S2. Most included individuals were born in England (83.5%), female (51.5%), and white (97.8%) (Table 1). At age 55, 27.4% had at least a university or equivalent degree, while 69.9% were married or lived with a partner. Although more men were overweight or obese (71.3%; *p* < 0.001), alcohol intake were higher among women (*p* < 0.001).

**Table 1:** Sociodemographic, behavioral, and clinical characteristics of the sample. *N* = 9137. United Kingdom, 2013

	Total, n (%)	Sex, n (%)		*p* value
		Male	Female	
**Sample, N**				–
0	17,415 (100.0)	9004 (52.5)	8410 (47.5)	
7 yr	15,425 (92.3)	7915 (51.3)	7506 (48.7)	
11 yr	15,333 (91.6)	7894 (51.4)	7449 (48.6)	
16 yr	14,650 (86.8)	7544 (51.5)	7106 (48.5)	
23 yr	12,533 (75.3)	6264 (50.0)	6269 (50,0)	
33 yr	11,465 (70.9)	5632 (49.1)	5833 (50.9)	
42 yr	11,416 (71.0)	5624 (49.3)	5792 (50.7)	
50 yr	9787 (61.9)	4819 (49.2)	4968 (50.8)	
55 yr	9137 (58.5)	4433 (48.5)	4704 (51.5)	
**Country of Birth**				0.771
England	7625 (83.5)	3695 (83.4)	3930 (83.6)	
Wales	482 (5.3)	236 (5.3)	246 (5.2)	
Scotland	826 (9.0)	396 (8.9)	430 (9.1)	
Not in Great Britain	204 (2.2)	106 (2.4)	98 (2.1)	
**Ethnicity, %**				**0.010**
White	8948 (97.9)	4344 (98.0)	4604 (97.9)	
Mixed	27 (0.3)	11 (0.2)	16 (0.3)	
Indian	33 (0.4)	11 (0.2)	22 (0.5)	
Pakistani/Bangladeshi	12 (0.1)	10 (0.3)	2 (0.1)	
Black	54 (0.6)	20 (0.5)	34 (0.7)	
Other	63 (0.7)	37 (0.8)	26 (0.5)	
**Educational level**^**a**^ **(*n*** **= 8952), %**				**< 0.001**
No academic qualification	1478 (16.5)	798 (18.4)	680 (14.7)	
CSE 2–5 or equivalent	1285 (14.3)	668 (15.4)	617 (13.4)	
O Level or equivalent	3021 (33.8)	1373 (31.6)	1648 (35.7)	
A level or equivalent	789 (8.8)	382 (8.8)	407 (8.8)	
University degree or equivalent	1998 (22.3)	910 (21.0)	1088 (23.6)	
Higher degree	381 (4.3)	207 (4.8)	174 (3.8)	
**Marital status**^**a**^ **(*n*** **= 9130), %**				**< 0.001**
Married/living with partner	6549 (71.7)	3.260 (73.6)	3.289 (69.9)	
Widowed	214 (2.3)	51 (1.2)	163 (3.5)	
Divorced/separated	1459 (16.0)	619 (14.0)	840 (17.9)	
Single	908 (10.0)	498 (11.2)	410 (8.7)	
**Income**^**a**^ **(£$) (*n*** **= 6459), %**				**< 0.001**
1st quintile (poorest)	1293 (20.0)	680 (20.3)	613 (19.7)	
2nd quintile	1317 (20.4)	555 (16.6)	762 (24.5)	
3rd quintile	1338 (20.7)	655 (19.5)	683 (22.0)	
4th quintile	1226 (19.0)	699 (20.9)	527 (16.9)	
5th quintile (wealthiest)	1287 (19.9)	761 (22.7)	526 (16.9)	
**BMI**^**a**^ **(*n*** **= 8483), %**				**< 0.001**
Normal	3041 (35.9)	1.212 (28.7)	1.829 (42.9)	
Overweight	3438 (40.5)	1.985 (47.1)	1.453 (34.1)	
Obese	2004 (23.6)	1.022 (24.2)	982 (23.0)	
**Number of units of alcohol in last 7 days**^**a**^ **(*n*** **= 8766), %**				**< 0.001**
1	1633 (18.1)	970 (22.2)	663 (14.3)	
2	2806 (31.1)	1495 (34.2)	1311 (28.2)	
3	1372 (15.2)	705 (16.1)	667 (14.3)	
4–6	2411 (26.7)	918 (21.0)	1.493 (32.1)	
7–8	800 (8.9)	282 (6.5)	518 (11.1)	
**Smoking**^**a**^ **(*n*** **= 9020), %**				0.071
Smoke cigarettes every day	1256 (13.9)	607 (13.9)	649 (14.0)	
Smokes occasionally/not everyday	297 (3.3)	141 (3.2)	156 (3.3)	
Ex-smoker	2879 (31.9)	1.451 (33.2)	1.428 (30.7)	
Never smoked cigarettes	4590 (50.9)	2.170 (49.7)	2.420 (52.0)	
**General health perception**^**a**^ **(*n*** **= 9039), %**				0.071
Excellent	1217 (13.5)	599 (13.7)	618 (13.3)	
Very good	3095 (34.2)	1531 (35.0)	1564 (33.5)	
Good	2915 (32.3)	1410 (32.2)	1505 (32.3)	
Fair	1266 (14.0)	605 (13.8)	661 (14.2)	
Poor	546 (6.0)	234 (5.3)	312 (6.7)	

^a^ At age 55. *BMI* Body mass index; Numbers in bold indicates statistical significance (*p* < 0.05)

Our analysis did not detect difference in LTPA between gender (*p* = 0.087), however, men reported higher work-related physical activity than women (*p* < 0.001). In addition, 80% of sample self-rated their health as good to excellent with no detectable difference between gender (*p* = 0.071).

Men were more physically active than women in age 11, 16, and 23 (*p* < 0.001; Table 2). The proportion of women who engage in in LTPA all days in the week was higher in age 33, 42, and 50. (*p* < 0.001).

**Table 2:** Leisure-time physical activity practice from age 7 to 55 among adults aged 55 years. United Kingdom, 2008

	Total, n (%)	Sex, n (%)		*p* value
		Male	Female	
**Childhood (7 yr)**	*N* = 8010 (87.7)			0.247
Inactive	321 (4.0)	148 (3.8)	173 (4.2)	
Normally active	6613 (82.6)	3187 (82.2)	3426 (82.9)	
Over active	1076 (13.4)	544 (14.0)	532 (12.9)	
**Childhood (11 yr)**	*N* = 7715 (84.4)			**< 0.001**
Hardly ever	866 (11.2)	332 (8.9)	534 (13.4)	
Sometimes	3332 (43.2)	1381 (37.0)	1951 (49.0)	
Most days	3517 (45.6)	2017 (54.1)	1500 (37.6)	
**Adolescence (16 yr)**				**< 0.001**
Inactive	8354 (57.0)	3948 (52.3)	4406 (62.0)	
Active	6296 (43.0)	3596 (47.7)	2700 (38.0)	
**Adult**				
**Young (23 yr)**	*N* = 7807 (85.4)			**< 0.001**
No sport last 4 weeks	3919 (50.2)	1439 (38.4)	2480 (61.1)	
Once last 4 weeks	529 (6.8)	272 (7.3)	257 (6.3)	
2–3 times last 4 weeks	764 (9.8)	410 (10.9)	354 (8.7)	
1–2 times a week	1495 (19.1)	833 (22.2)	662 (16.3)	
3–4 times per week	607 (7.8)	442 (11.8)	165 (4.1)	
5 or more times per week	493 (6.3)	352 (9.4)	141 (3.5)	
**Young (33 yr)**	*N* = 7918 (86.7)			**< 0.001**
No exercise	1648 (20.8)	764 (20.3)	884 (21.3)	
Less often	227 (2.9)	122 (3.2)	105 (2.5)	
2–3 times a month	508 (6.4)	261 (6.9)	247 (6.0)	
once a week	1808 (22.8)	824 (21.8)	984 (23.7)	
2–3 days a week	1752 (22.1)	915 (24.3)	837 (20.2)	
4–5 days a week	519 (6.6)	298 (7.9)	221 (5.4)	
Every day	1456 (18.4)	589 (15.6)	867 (20.9)	
**Middle (42 yr)**	*N* = 8433 (92.3)			**< 0.001**
No exercise	2058 (24.4)	914 (22.6)	1144 (26.1)	
Less often	210 (2.5)	102 (2.5)	108 (2.5)	
2–3 times a month	536 (6.4)	304 (7.5)	232 (5.3)	
Once a week	1587 (18.8)	832 (20.6)	755 (17.2)	
2–3 days a week	1847 (21.9)	904 (22.3)	943 (21.5)	
4–5 days a week	805 (9.5)	407 (10.1)	398 (9.0)	
Every day	1390 (16.5)	581 (14.4)	809 (18.4)	
**Older (50 yr)**	*N* = 8362 (91.5)			**< 0.001**
None	1849 (22.1)	858 (21.1)	991 (23.1)	
3 times per month or less	633 (7.5)	328 (8.1)	305 (7.1)	
Once a week	1269 (15.2)	678 (16.7)	591 (13.7)	
2–3 days a week	1899 (22.7)	981 (24.2)	918 (21.3)	
4–5 days a week	900 (10.8)	430 (10.6)	470 (10.9)	
Every day	1812 (21.7)	786 (19.3)	1026 (23.9)	
**Older (55 yr)**	*N* = 9011 (98.6)			0.087
Never	943 (10.5)	452 (10.4)	491 (10.6)	
Less than once per month	1111 (12.3)	570 (13.1)	541 (11.6)	
At least once per month	1227 (13.6)	565 (12.9)	662 (14.2)	
At least once per week	5730 (63.6)	2776 (63.6)	2954 (63.6)	

Numbers in bold indicates statistical significance (*p* < 0.05)

From the 8414 participants who reported data about all included morbidities at age 55, 2278 (33.0%) were classified as having multimorbidity. Multimorbidity was more likely among participants with low educational achievement, at lowest quintile interval of income, who were not regular drinkers of alcoholic beverages, and with worse general health perception, as shown in Supplementary Table S3.

Table 3 exhibits results from log-likelihood ratio test to examine which life course model best described the association between LTPA through the life course and multimorbidity. We compared the fit of a series of nested logistic models (critical, accumulation or sensitive period models) with a fully saturated model. Critical periods of LTPA during childhood and adulthood provided an inferior fit compared to the saturated model (*p* < 0.001). However, critical period model for LTPA during adolescence (*p* > 0.99) and sensitive model (*p* > 0.99) provided a fit not worse than saturated model with no clear difference between models based on *p*-value, log-likelihood, and AIC. Accumulation models provided similar fit compared to saturated model (*p* > 0.99); however, AIC values were higher than observed in adolescence as a critical period and the sensitive model, as shown in Table 3. Therefore, adolescence as a critical period and the sensitive models were chosen as the two simplest models that best explained the association between LTPA through life stages and the risk of multimorbidity at age 55.

**Table 3:** Likelihood-ratio test to estimate the best model of physical activity throughout life course and the risk of multimorbidity at age 55. National Child Development Study. United Kingdom (1958–2013). *N* = 8414

	LL	AIC	*p*-value ^a^
No effect	− 5337.0	10,675.9	< 0.001
Critical period			
Childhood	− 2968.6	4933.1	< 0.001
**Adolescence**	**− 1781.1**	**3000.1**	**> 0.99**
Young-adult	− 2225.7	3733.6	< 0.001
Middle-aged adults	− 2251.9	3782.2	< 0.001
Middle-to-older adults	− 2253.8	3779.4	< 0.001
Accumulation			
Categorical	− 1650.7	3317.2	> 0.99
Continuous	−1650.7	3310.3	> 0.99
**Sensitive period**	**−1781.1**	**3000.1**	**> 0.99**
Saturated model	− 2024.1	3340.4	^b^

*LL* log-likelihood, *AIC* Akaike information criterion

^a^ Compared to saturated model (all possible exposure combinations and interactions describes all possible trajectories of physical activity throughout life course);

^b^ *p* value not applicable

As shown in Table 4, LTPA during adolescence was associated with lower odds for multimorbidity at age 55 (OR: 0.82; 95%CI: 0.70; 0.97) independently of LTPA performed in other stages of life. Based on the sensitive model, being physically active during leisure time at adolescence (OR: 0.83; 95%CI: 0.70; 0.98) and middle-to-late adult life (ages 50 and 55) (OR:0.82; 95%CI: 0.69; 0.98) also promoted a protective effect against multimorbidity at age 55.

**Table 4:** Odds ratio and 95% confidence interval (CI) for multimorbidity at age 55 by different life course models

	Model 1			Model 2			Model 3		
	OR	95% CI	*p* value	OR	95% CI	*p* value	OR	95% CI	*p* value
Saturation model									
0,0,0,0,0	1.01	0.32, 1.01	0.054	1.68	0.45, 1.68	0.678	1.99	0.45, 1.99	0.896
0,0,0,1,0	2.29	0.67, 2.29	0.504	3.81	0.93, 3.81	0.081	**5.25**	**1.10, 5.25**	**0.027**
0,0,1,0,0	**0.95**	**0.32, 0.95**	**0.031**	1.31	0.37, 1.31	0.266	1.37	0.33, 1.37	0.272
0,0,1,0,1	1.51	0.53, 1.51	0.682	2.38	0.7, 2.38	0.416	2.04	0.50, 2.04	0.975
0,0,1,1,0	**0.80**	**0.29, 0.8**	**0.005**	1.31	0.4, 1.31	0.284	1.70	0.45, 1.70	0.698
0,0,1,1,1	1.44	0.51, 1.44	0.553	2.49	0.74, 2.49	0.323	3.14	0.80, 3.14	0.184
0,1,1,0,0	**0.79**	**0.35, 0.79**	**0.002**	1.33	0.51, 1.33	0.420	1.71	0.58, 1.71	0.981
0,1,1,0,1	1.84	0.47, 1.84	0.835	2.75	0.52, 2.75	0.676	3.03	0.48, 3.03	0.698
0,1,1,1,1	1.06	0.24, 1.06	0.070	1.84	0.33, 1.84	0.574	2.21	0.33, 2.21	0.739
1,0,0,0,0	2.11	0.24, 2.11	0.546	3.02	0.1, 3.02	0.499	2.40	0.07, 2.40	0.312
1,0,0,0,1	**0.85**	**0.2, 0.85**	**0.017**	1.82	0.37, 1.82	0.622	2.87	0.49, 2.87	0.703
1,0,0,1,0	1.59	0.47, 1.59	0.645	2.34	0.58, 2.34	0.664	2.40	0.49, 2.40	0.841
1,0,0,1,1	**0.83**	**0.27, 0.83**	**0.009**	1.39	0.38, 1.39	0.333	1.74	0.40, 1.74	0.631
1,0,1,0,0	**0.88**	**0.28, 0.88**	**0.016**	1.28	0.33, 1.28	0.210	1.59	0.34, 1.59	0.440
1,0,1,0,1	**0.66**	**0.28, 0.66**	**< 0.001**	1.05	0.39, 1.05	0.074	1.45	0.47, 1.45	0.516
1,0,1,1,0	1.83	0.57, 1.83	0.933	2.94	0.75, 2.94	0.260	2.60	0.55, 2.60	0.648
1,0,1,1,1	1.47	0.39, 1.47	0.419	2.34	0.51, 2.34	0.825	3.59	0.66, 3.59	0.321
1,1,0,0,0	1.82	0.37, 1.82	0.622	3.61	0.58, 3.61	0.421	4.84	0.61, 4.84	0.307
1,1,0,0,1	1.45	0.46, 1.45	0.496	2.20	0.59, 2.2	0.695	3.05	0.71, 3.05	0.304
1,1,0,1,0	1.33	0.45, 1.33	0.351	1.99	0.55, 1.99	0.882	2.50	0.6, 2.5	0.580
1,1,0,1,1	**0.95**	**0.32, 0.95**	**0.032**	1.29	0.36, 1.29	0.245	1.82	0.44, 1.82	0.760
1,1,1,0,0	**1.00**	**0.33, 1**	**0.049**	1.58	0.44, 1.58	0.587	2.06	0.49, 2.06	0.987
1,1,1,0,1	**0.90**	**0.39, 0.9**	**0.013**	1.34	0.51, 1.34	0.438	1.92	0.64, 1.92	0.713
1,1,1,1,0	2.29	0.57, 2.29	0.703	2.69	0.56, 2.69	0.608	3.25	0.56, 3.25	0.507
Critical period									
Childhood	1.01	0.91; 1.12	0.877	0.96	0.85; 1.09	0.526	0.97	0.84; 1.13	0.744
Adolescence	0.90	0.81; 1.00	0.052	0.88	0.77; 1.01	0.081	**0.82**	**0.70; 0.97**	**0.023**
Young-adult	**0.74**	**0.66; 0.84**	**< 0.001**	**0.82**	**0.71; 0.95**	**0.008**	0.94	0.79; 1.12	0.497
Middle-aged adults	**0.77**	**0.69; 0.85**	**< 0.001**	**0.85**	**0.76; 0.96**	**0.009**	0.90	0.77; 1.03	0.137
Middle-to-older adults	**0.64**	**0.57; 0.70**	**< 0.001**	**0.69**	**0.61; 0.78**	**< 0.001**	**0.85**	**0.73; 0.98**	**0.027**
Accumulation									
Categorical									
None	Ref			Ref			Ref		
One	0.48	0.21; 1.07	0.072	0.56	0.22; 1.39	0.210	0.69	0.23; 2.08	0.513
Two	**0.45**	**0.21; 0.97**	**0.041**	0.56	0.23; 1.32	0.183	0.69	0.24; 1.96	0.483
Three	**0.38**	**0.17; 0.81**	**0.012**	0.47	0.20; 1.11	0.177	0.65	0.23; 1.84	0.420
Four	**0.32**	**0.15; 0.67**	**< 0.001**	**0.39**	**0.17; 0.91**	**0.030**	0.57	0.21; 1.61	0.291
Five	**0.29**	**0.14; 0.63**	**< 0.001**	**0.33**	**0.14; 0.78**	**0.011**	0.52	0.18; 1.46	0.213
Continuous	**0.86**	**0.81; 0.91**	**< 0.001**	**0.85**	**0.79; 0.91**	**< 0.001**	**0.91**	**0.84; 0.99**	**0.001**
Sensitive period									
Childhood	1.00	0.91; 1.12	0.877	0.96	0.81; 1.15	0.683	0.98	0.80; 1.20	0.848
Adolescence	0.90	0.81; 1.00	0.052	**0.81**	**0.67; 0.99**	**0.041**	**0.83**	**0.70, 0.98**	**0.025**
Young-adult	**0.74**	**0.66; 0.84**	**< 0.001**	0.87	0.70; 1.07	0.187	0.98	0.77; 1.25	0.265
Middle-aged adults	**0.77**	**0.69; 0.84**	**< 0.001**	0.91	0.75; 1.09	0.305	0.90	0.72; 1.12	0.338
Middle-to-older adults	**0.63**	**0.57; 0.70**	**< 0.001**	**0.69**	**0.59; 0.79**	**< 0.001**	**0.82**	**0.69; 0.98**	**0.025**

Numbers in bold indicates statistical significance (*p* < 0.05)

Each number position (“0” for inactive or “1” for active) for the saturation model represents, respectively, 5 lifetime stages: childhood, adolescence, and young, middle, and middle-to-older adults

Model 1: Unadjusted

Model 2: Adjusted for sex, education, income, marital status, country of birth, ethnicity

Model 3: Model 2 + smoking, alcohol, and hours of sleep at age 50

## Discussion

Our findings confirmed our hypothesis that engage in LTPA through life would reduce the risk for multimorbidity in older ages. Both sensitive and critical periods best explained the association between LTPA through life course and multimorbidity. We revealed that being physically active at adolescence and middle-to-late adult life reduced in about 17 and 18% the odds of multimorbidity at age 55, respectively, even when controlled for LTPA at all other analyzed ages. Similarly, engage in LTPA early in life was revealed as a critical period for decreasing the odds of multimorbidity at age 55.

Adolescence is an important life period where healthy lifestyle promotion (e.g. LTPA) could reduce the risk for some conditions such as obesity [23, 24], diabetes [25], and cognitive impairment [26] in older ages. Nevertheless, physical inactivity is predominant among children and adolescents aged between 6 and 15 years [27–29]. This scenario seems to be associated with additional health-related events over the next decades, turning out also to be related to the prevalence of multimorbidity [11]. Given the burden on healthcare systems, that are already poised by elevated prevalence of those conditions [2], it is important to consider physical activity as a continued lifetime strategy for disease prevention instead of a solely rehabilitative method at advanced ages.

In a retrospective study conducted by Fernandes and Zanesco [25], physical activity at childhood (7 to 10 years) and adolescence (11 to 17 years) were related to a decreased risk for arterial hypertension and diabetes in adulthood (18 or more years). In our study, we observed that LTPA at adolescence was associated with odds reduction for multimorbidity at age 55 in the final adjusted model. The fact that this association was statistically significant in model 3 (adjusted for smoking, alcohol intake, and hours of sleep at middle-to-late adult life) show the importance of adolescence as a critical period for reducing the risk of multimorbidity in later life. Taken together, these previous findings support the notion of a protective physical activity “legacy” at early ages of childhood against multimorbidity at older ages. As this population is on school ages, the development of multicomponent school-based interventions promoting healthy lifestyle should be encouraged in order to reduce the likelihood of being affected by multimorbidity [30]. This scenario could also result in an increased quality of life and decreased the burden of multimorbidity in healthcare systems [31, 32].

Physical activity engagement in practice adulthood has been associated with reduced risk for chronic diseases and all-cause mortality [13]. Based on data from the English Longitudinal Study of Aging, Hamer et al. [15] reported that from a sample of older adults aged 50 or more, those who were physically inactive at baseline and became active at older ages had higher odds to have healthier aging than those who remained inactive. Similarly, we reported that LTPA at middle-to-late adult life (ages 50 and 55) had a significant protective effect against multimorbidity at age 55. Although the World Health Organization [33] recommends 150 min per week of moderate-to-vigorous physical activity, some studies have shown that lower levels could lead to reduced risk for chronic diseases and all-cause mortality. In this regard, Ekelund et al. [34, 35] revealed that lower doses of moderate-to-vigorous physical activity (i.e., 24 min per day) could contribute to the risk reduction of premature mortality, with a dose-response pattern in adulthood (20 years or more).

Dregan et al. [26] reported that LTPA sustained through life periods improved cognitive function in older adults. Another study [15] showed that becoming physically active at age 50 was associated with a lower risk for depression symptoms, cognitive impairment, and functional disability at older ages (8-year follow-up). Even though our results corroborate previous findings, cumulative models did not provide a sufficient explanation of the effect of LTPA during life course in multimorbidity. However, we highlight that, whenever feasible, LTPA must be promoted in all age groups especially among those groups with a higher prevalence of physical inactivity (children, adolescents, and older adults) [27, 36, 37]. Interventions tailored from those groups are necessary in order to prevent in the future a scenario with an even higher prevalence of multimorbidity and its burden on the healthcare system [38–41].

Some limitations of this present study must be acknowledged. First, LTPA was measured by questionnaires. In order to reduce the bias from that measurement, we chose to use in our study only those sweeps with face-to-face interview administrated questionnaire. The 1958 National Child Development Survey is one of the oldest national-based cohort studies, so although the level of LTPA was not examined by devices-based measurements, the information from those sweeps are reliable, comparable, and relevant [20]. Also, as the instrument does not provide details about the weekly volume of LTPA, it was not possible to categorize participants based on the World Health Organization’s guidelines of physical activity. However, the operationalization for LTPA to categorize individuals as physically active or inactive was based on previously published studies from the same birth cohort [20, 42]. Second, participants with multimorbidity were more likely to have no academic qualification and be at the lowest quintile of income, so residual confounding cannot be ruled out. Also, there were no data available on the participants’ family history regarding chronic diseases. Third, although the loss to follow-up may be interpreted as a source of selection bias, the NCDS cohort remains largely representative of the sample that it was drawn with a response rate of sweep (58.5%) [43]. Furthermore, we used multiple imputations to avoid further reductions in the sample due to missing information [22]. Four, although data from other chronic diseases such as stroke were not available, the chronic conditions we considered to identify multimorbidity were highly prevalent among adults aged between 50 and 54 years-old in the United Kingdom in 2019 such as low back pain (18.2%), hearing impairment (17.5%), and diabetes (13.2%) [44]. Fifth, reversal causality cannot be ruled out especially regarding the protective effect of LTPA at age 55 against multimorbidity at the same age.

## Conclusions

In conclusion, we identified LTPA during adolescence as a critical period associated with reduced risk for multimorbidity in late adult life. Similar protective status was found in the sensitive model for LTPA during the same period and in middle-to-late adulthood (ages 50 and 55). Although analysis of factors associated with the outcome along lifetime is complex and should be carefully interpreted, our results support the need for LTPA promotion through intervention tailored especially on schooling age and older ages to reduce the burden of multimorbidity.

## Supplementary Information

**Additional file 1: Figure S1.** Flow diagram about sampling process through sweeps. 1958 National Child Development Study. United Kingdom.

**Additional file 2: Table S1.** Operationalization of physical activity since age 7 sweep and in the present study.

**Additional file 3: Table S2.** Sociodemographic, behavioral, and clinical characteristics from the included and excluded sample. *N* = 18.558. United Kingdom, 2013.

**Additional file 4: Table S3.** Sociodemographic, behavioral, and clinical characteristics from the sample. *N* = 8414. United Kingdom, 2013.

## Data Availability

The datasets generated and/or analyzed during the current study are available in the Data Archive at the University of Essex, https://beta.ukdataservice.ac.uk/datacatalogue/studies/study?id=5560

## Abbreviations

LTPA: Leisure-time physical activity
NCDS: National Child Development Study
