# Supplementary material for: Multimorbidity and leisure-time physical activity over the life course: a population-based birth cohort study
Source: BMC Public Health. 2021 Apr 9;21:700. doi: 10.1186/s12889-021-10719-7 (PMC8033277; doi:10.1186/s12889-021-10719-7)
Supplement: Supplementary file 1 — Additional file 1: Figure S1. Flow diagram about sampling process through sweeps. 1958 National Child Development Study. United Kingdom. [file 12889_2021_10719_MOESM1_ESM.docx]

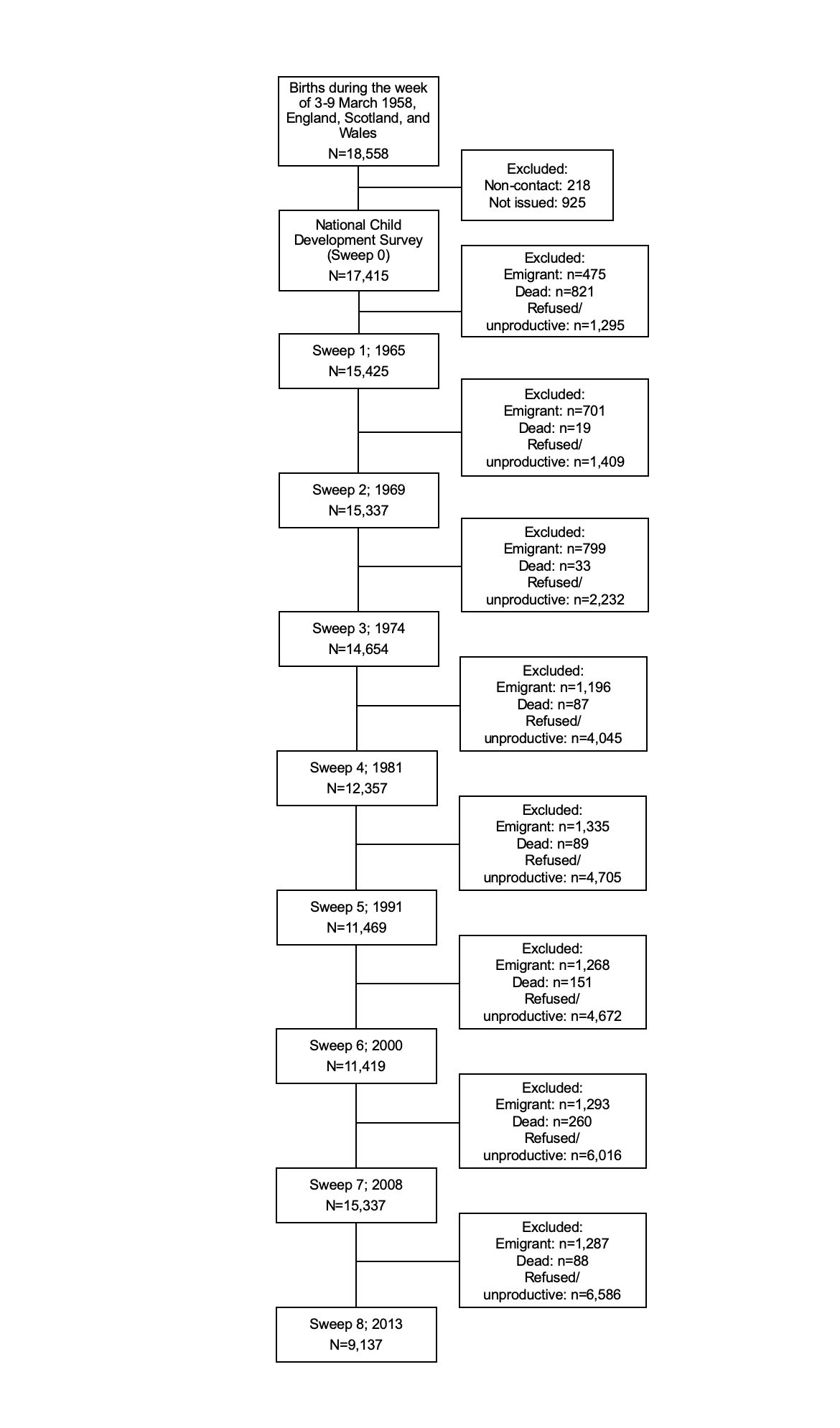


Supplementary Figure S1**.** Flow diagram about sampling process through sweeps. 1958 National Child Development Study. United Kingdom.
