# Supplementary material for: Multimorbidity and leisure-time physical activity over the life course: a population-based birth cohort study
Source: BMC Public Health. 2021 Apr 9;21:700. doi: 10.1186/s12889-021-10719-7 (PMC8033277; doi:10.1186/s12889-021-10719-7)
Supplement: Supplementary file 2 — Additional file 2: Table S1. Operationalization of physical activity since age 7 sweep and in the present study. [file 12889_2021_10719_MOESM2_ESM.docx]

Supplementary Table S1. Operationalization of physical activity since age 7 sweep and in the present study.

| Sweep | Responder | Question/Instrument | Categories | |
| --- | --- | --- | --- | --- |
|  |  |  | NCDS | Present study* |
| Age 7 sweep | Mother | Question: Is the child normally active, inactive and quiet (prefer to sit and watch), or restless and overactive (can’t keep still)? | Inactive; Normally; active; Over active | Inactive; Normally active + Over active |
| Age 11 sweep | Individual | Read each one carefully and decide whether  you do It often (nearly every day), sometimes; never or hardly ever | Never or hardly ever; sometimes;  Often (nearly every day) | Never or hardly ever + sometimes;  Often (nearly every day) |
| Age 16 sweep | Individual | Below is a list of four things which many people do in their spare time. Please show by ringing one of the numbers for each one whether this is something that you do often, sometimes, never or hardly never. | Never or hardly ever; sometimes;  Often | Often was scored as 2, sometimes scored as 1, never and not available were scored as 0. The scores were summed across the variables and the resulting categories collapsed to 4. The two most and least active categories were merged as active and inactive, respectively. |
| Age 23 sweep | Individual | Please tell me from this card, how often you have done each one over the past 4 weeks. | Not at all in the last 4 weeks; once in the last 4 weeks; 2 or 3 times in the last 4 weeks; once or twice per week; 3 or 4 times per week; 5 times per week | Inactive (less than once per week); active (once per week or more) |
| Age 33 sweep | Individual | How often do you take part in any activity of this type? | Less often; 2-3 times per month; once a week; 2-3 days a week; 4-5 days a week; every day or most days | Inactive: less than once a week;  Active: once a week or more |
| Age 42 sweep | Individual | How often do you take part in any activity of this type? | Less often; 2-3 times per month; once a week; 2-3 days a week; 4-5 days a week; every day | Inactive: less than once a week;  Active: once a week or more |
| Age 50 sweep | Individual | How often do you take part in any activity of this type? | Less often; 2-3 times per month; once a week; 2-3 days a week; 4-5 days a week; every day | Inactive: less than once a week;  Active: once a week or more |
| Age 55 sweep | Individual | How often do you take part in any activity of this type? | Less often; 2-3 times per month; once a week; 2-3 days a week; 4-5 days a week; every day | Inactive: less than once a week;  Active: once a week or more |

* We re-categorized physical activity only for logistic regression analyses about number of life stages being physically active and number of morbidities at age 55 (Table 4).
