# Supplementary material for: Multimorbidity and leisure-time physical activity over the life course: a population-based birth cohort study
Source: BMC Public Health. 2021 Apr 9;21:700. doi: 10.1186/s12889-021-10719-7 (PMC8033277; doi:10.1186/s12889-021-10719-7)
Supplement: Supplementary file 3 — Additional file 3: Table S2. Sociodemographic, behavioral, and clinical characteristics from the included and excluded sample. N = 18.558. United Kingdom, 2013. [file 12889_2021_10719_MOESM3_ESM.docx]

Supplementary Table S2. Sociodemographic, behavioral, and clinical characteristics from the included and excluded sample. N=18.558. United Kingdom, 2013.

|  | Excluded (n=9,424) | Included (n=9,137) | p value |
| --- | --- | --- | --- |
| **Sex** |  |  | **<0.001** |
| Male | 5,165 (54.8) | 4,433 (48.5) |  |
| Female | 4,256 (45.2) | 4,704 (51.5) |  |
| **Country of Birth** |  |  | **<0.001** |
| England | 7,221 (76.7) | 7,304 (80.0) |  |
| Wales | 451 (4.8) | 463 (5.1) |  |
| Scotland | 1,085 (11.5) | 905 (9.9) |  |
| Great Britain | 162 (1.7) | 168 (1.8) |  |
| Not in Great Britain | 502 (5.3) | 297 (3.2) |  |
| **Race, %** |  |  | **<0.001** |
| White | 9,111 (96.7) | 8.945 (97.9) |  |
| Mixed | 41 (0.4) | 27 (0.3) |  |
| Indian | 64 (0.7) | 33 (0.4) |  |
| Pakistani/Bangladeshi | 15 (0.2) | 12 (0.1) |  |
| Black | 116 (1.2) | 54 (0.6) |  |
| Other | 74 (0.8) | 66 (0.7) |  |
| **Educational level (n=9.780)*, %** |  |  | **<0.001** |
| No academic qualification | 523 (36.8) | 1,387 (16.6) |  |
| CSE 2-5 or equivalent | 418 (29.4) | 2,857 (34.2) |  |
| A level or equivalent | 563 (9.3) | 172 (7.7) |  |
| University degree or equivalent | 170 (12.0) | 1,834 (21.9) |  |
| Higher degree | 26 (1.8) | 330 (3.9) |  |
| **Marital status* (n=5,755), %** |  |  | **<0.001** |
| Married/living with partner | 400 (62.2) | 3,725 (72.9) |  |
| Widowed | 11 (1.7) | 69 (1.3) |  |
| Divorced/separated | 159 (24.7) | 838 (16.4) |  |
| Single | 73 (11.3) | 479 (9.4) |  |
| **Economic activity* (n=9,758), %** |  |  | **<0.001** |
| Employee | 836 (59.6) | 6,084 (72.8) |  |
| Self-employed | 211 (15.0) | 1,128 (13.5) |  |
| Unemployed | 232 (16.5) | 569 (6.8) |  |
| Caregiver | 95 (6.8) | 426 (5.1) |  |
| Other | 29 (16.5) | 569 (6.8) |  |
| **BMI* (n=4,956), %** |  |  | **0.008** |
| Normal | 146 (30.7) | 1,545 (34.5) |  |
| Overweight | 179 (37.6) | 1,806 (40.3) |  |
| Obese | 151 (31.7) | 1,129 (25.5) |  |
| **Number of units of alcohol in last 7 days* (n=7.772), %** |  |  | **<0.001** |
| 1-2 | 551 (45.4) | 4,086 (52.6) |  |
| 3-4 | 387 (31.9) | 2,390 (30.7) |  |
| 5-6 | 182 (15.0) | 913 (11.7) |  |
| 7+ | 94 (7.7) | 383 (4.9) |  |
| **Smoking* (n=8,342), %** |  |  | **<0.001** |
| Never | 550 (39.5) | 3.958 (47.5) |  |
| Ex-smoker | 341 (24.5) | 2,677 (32.1) |  |
| Current smoker | 500 (35.9) | 1,707 (20.5) |  |

* At age 50. BMI: Body mass index; Numbers in bold indicates statistical significance (p<0.05).
