# Supplementary material for: Multimorbidity and leisure-time physical activity over the life course: a population-based birth cohort study
Source: BMC Public Health. 2021 Apr 9;21:700. doi: 10.1186/s12889-021-10719-7 (PMC8033277; doi:10.1186/s12889-021-10719-7)
Supplement: Supplementary file 4 — Additional file 4: Table S3. Sociodemographic, behavioral, and clinical characteristics from the sample. N = 8414. United Kingdom, 2013. [file 12889_2021_10719_MOESM4_ESM.docx]

Supplementary Table S3. Sociodemographic, behavioral, and clinical characteristics from the sample. N=8,414. United Kingdom, 2013.

|  | Multimorbidity, n (%) | | p value |
| --- | --- | --- | --- |
|  | No (n=5,636) | Yes (n=2,778) |  |
| **Sex** |  |  | 0.656 |
| Male | 2,793 (49.6) | 1,391 (50.1) |  |
| Female | 2,843 (50.4) | 1,387 (49.9) |  |
| **Country of Birth** |  |  | 0.077 |
| England | 4,556 (80.8) | 2,187 (78.7) |  |
| Wales | 258 (4.6) | 160 (5.8) |  |
| Scotland | 531 (9.4) | 290 (10.4) |  |
| Great Britain | 107 (1.9) | 49 (1.8) |  |
| Not in Great Britain | 184 (3.3) | 92 (3.3) |  |
| **Race, %** |  |  | 0.075 |
| White | 5,536 (98.2) | 2,707 (97.4) |  |
| Mixed | 11 (0.2) | 13 (0.5) |  |
| Indian | 16 (0.3) | 13 (0.5) |  |
| Pakistani/Bangladeshi | 8 (0.1) | 3 (0.1) |  |
| Black | 33 (0.6) | 17 (0.6) |  |
| Other | 32 (0.6) | 25 (0.9) |  |
| **Educational level* (n=7,735), %** |  |  | **<0.001** |
| No academic qualification | 716 (13.8) | 537 (21.0) |  |
| CSE 2-5 or equivalent | 1,789 (34.5) | 875 (34.3) |  |
| O Level or equivalent | 701 (13.5) | 397 (15.5) |  |
| A level or equivalent | 490 (9.5) | 206 (8.1) |  |
| University degree or equivalent | 1,245 (24.0) | 468 (18.3) |  |
| Higher degree | 241 (4.7) | 70 (2.7) |  |
| **Marital status* (n=8,408), %** |  |  | **<0.001** |
| Married/living with partner | 4,157 (73.8) | 1,940 (69.9) |  |
| Widowed | 110 (1.9) | 85 (3.1) |  |
| Divorced/separated | 843 (15.0) | 478 (17.2) |  |
| Single | 521 (9.3) | 274 (9.9) |  |
| **Income* (£$), (n=6,151) %** |  |  | **<0.001** |
| 1^st^ quintile (poorest) | 654 (16.1) | 550 (26.2) |  |
| 2^nd^ quintile | 752 (18.6) | 484 (23.1) |  |
| 3^rd^ quintile | 893 (22.0) | 389 (18.5) |  |
| 4^th^ quintile | 836 (20.6) | 347 (16.5) |  |
| 5^th^ quintile | 916 (22.6) | 330 (15.7) |  |
| **BMI*, %** |  |  | **<0.001** |
| Normal | 1,309 (41.7) | 185 (16.7) |  |
| Overweight | 1,348 (43.0) | 371 (33.5) |  |
| Obese | 479 (15.3) | 550 (49.7) |  |
| **Number of units of alcohol in last 7 days* (n=8,189), %** |  |  | **<0.001** |
| 0 | 1,428 (26.0) | 1,099 (40.7) |  |
| 1-3 | 957 (17.4) | 370 (13.7) |  |
| 4-6 | 890 (16.2) | 307 (11.4) |  |
| 7+ | 2,211 (40.3) | 927 (34.3) |  |
| **Smoking* (n=7,719), %** |  |  | **<0.001** |
| Never | 2,553 (48.9) | 1,132 (44.5) |  |
| Ex-smoker | 1,643 (31.7) | 848 (33.3) |  |
| Current smoker | 999 (19.3) | 564 (22.2) |  |
| **General health perception*** |  |  | **<0.001** |
| Excellent | 1,065 (18.9) | 103 (3.7) |  |
| Very good | 2,344 (41.6) | 586 (21.1) |  |
| Good | 1,694 (30.1) | 1,007 (36.3) |  |
| Fair | 447 (7.9) | 699 (25.2) |  |
| Poor | 85 (1.5) | 381 (13.7) |  |

* At age 55. BMI: Body mass index; Numbers in bold indicates statistical significance (p<0.05).
